# Development, Acceptability, and Feasibility of a Digital Module for Coping with COVID-19 Distress: Pragmatic Retrospective Study

**DOI:** 10.1089/tmr.2021.0013

**Published:** 2021-07-15

**Authors:** Monica S. Wu, Jocelyn Lau, Chelsey Wilks, Connie Chen, Anita Lungu

**Affiliations:** ^1^ Lyra Health, Burlingame, California, USA.; ^2^ Department of Psychological Sciences, University of Missouri-St. Louis, St. Louis, Missouri, USA.

**Keywords:** blended care, cognitive-behavioral, COVID, digital

## Abstract

***Background:*** The coronavirus disease 2019 (COVID-19) pandemic and the measures to help contain it have taken a significant toll on mental health. Blended care psychotherapy combining provider-led care with digital tools can help alleviate this toll. This study describes the development of digital activities designed to teach cognitive-behavioral skills for coping with COVID-19 distress, and evaluates initial acceptability and feasibility data.

***Materials and Methods:*** Using a pragmatic retrospective cohort design, data from 664 U.S.-based individuals enrolled in blended care psychotherapy were analyzed. Descriptive analyses summarized acceptability for the digital activities. Ordinal logistic regression analyses were conducted on a subsample (*n* = 162) to explore the association between clients' attitudes toward the digital lesson and reported practice of skills in the exercise.

***Results:*** The majority of clients completed the assigned digital lesson and exercise. Clients reported finding the lesson valuable and relevant for coping with COVID-19 distress, and they intended to apply the skills to their lives. Higher agreement with these attitude questions was associated with a significantly greater number of skills practiced on the digital exercise.

***Discussion:*** Clients who were assigned a cognitive-behaviorally oriented digital lesson and/or exercise within a blended care model largely engaged with the materials and found them valuable. Clients with more positive attitudes about the digital lesson reported using more coping skills.

***Conclusions:*** Digital modules that teach specific skills for coping with COVID-19 can be integrated into treatment and minimize provider burden. Future study should investigate the clinical impact of these digital activities on psychiatric symptoms and personalizing the content.

## Introduction

The coronavirus disease 2019 (COVID-19) is one of most pervasive and consequential pandemics on record, with multiple waves having already occurred. State and local governments have implemented widespread policies designed to reduce spread and infection of COVID-19 including mandatory lockdowns, business closures, and work-from-home policies.^1^ The dynamic nature of this pandemic has bred high levels of uncertainty, leading to widespread increases in distress.^2^ Although the full impact of COVID-19 on mental health is yet to be fully known, emerging research suggests population-level increases in anxiety, depression,^3–5^ and suicidal ideation.^6^ Consequently, it is imperative to identify, adapt, and disseminate psychological interventions that can address these evolving mental health care needs.

Evidence-based psychotherapies, such as cognitive-behavioral therapy (CBT), have been effective in addressing a wide range of mental health concerns,^7^ including those exacerbated by the COVID-19 pandemic.^8^ However, there are various barriers to scaling evidence-based psychotherapies in response to increased mental health care needs, especially within the context of a prolonged pandemic. First, even before COVID-19, the number of mental health providers delivering evidence-based psychotherapies was insufficient to meet the need for care, with less than half of adults with mental illness receiving treatment in 2019.^9^ Second, disseminating evidence-based interventions in a scalable manner without compromising quality have been a long-standing barrier to care.^10^ Third, psychotherapists are at high risk of burnout and compassion fatigue.^11–13^ In a pandemic, this risk is further compounded since therapists are trying to support clients, while experiencing the same pandemic-related difficulties themselves. This highlights the need to find a way to concurrently reduce provider burden and promote increased coping skills in the client.

Psychological interventions deliver the most value if they can be quickly disseminated on a large scale, while reducing the burden added onto mental health providers. In recent years, technology has played a key role in facilitating this process. Specifically, internet-administered CBT has been found to be clinically effective, while being automated and scalable.^14^ Similarly, mobile apps allow expedited access to a variety of skills, and have even been adapted to address distress related to the COVID-19 pandemic.^15^ However, these intervention modalities often have high drop-out rates and low adherence,^16,17^ suggesting the need for increased support and follow-up. One possible solution to effectively address these needs is blended care psychotherapy.

In blended care psychotherapy, individual sessions with a therapist are combined with personalized digital materials that teach skills at scale.^18^ Therefore, blended care CBT (BC-CBT) programs retain the human connection and personalization inherent to traditional care with a therapist, with the added benefit of increased accessibility of online CBT content. BC-CBT delivered through teletherapy can represent a more scalable solution for delivering psychological support during periods of high need, such as during the COVID-19 pandemic.^19^

There is a paucity of research on how to develop and disseminate digital interventions that address pandemic-related distress, especially within the context of a BC-CBT program. To address this gap, this study describes the development process of a digital module, consisting of an educational video lesson and a practice exercise, to teach CBT-based skills for COVID-19–related distress. Initial feasibility and acceptability data on this digital module will be evaluated. Furthermore, clients who report more positive attitudes toward the video lesson are hypothesized to report a greater use of coping skills, given that higher treatment expectations have been linked to greater homework compliance and skills use.^20,21^ Collectively, these data highlight the utility of COVID-19–specific digital tools in real-world settings, and can inform the development and dissemination of such content within blended care programs.

## Methods

### Study design and participants

This study utilizes deidentified data collected from participants in Lyra Clinical Associates P.C.'s BC-CBT program. The study participants were clients who resided in the United States and had access to this treatment program as a mental health benefit offered by Lyra Health, Inc., which contracts with self-insured employers to deliver mental health benefit programs, and is partnered with Lyra Clinical Associates to deliver the clinical services available under the program to eligible employees and their dependents.

All therapy activities were conducted through a secure Health Insurance Portability and Accountability Act (HIPAA)-compliant online platform proprietary to Lyra Health, which was accessible through any device with a browser (e.g., laptop and smartphone). Individuals enrolled in BC-CBT attended sessions and were assigned digital activities, and the platform recorded activity assignments and completions. In addition, clients answered questions about the relevance and usefulness of these digital activities. Specifically, clients' attitudes were measured through their reported relevance, intent to practice, and value of the digital activity. This pragmatic retrospective analysis of deidentified data gathered from treatment offered by Lyra Clinical Associates was determined to be not human subjects research by the Western IRB.^22^

To be included in this study, clients must have been ≥18 years of age and attended at least one therapy session with a BC-CBT therapist between May 7, 2020, and October 15, 2020. More detailed inclusion and exclusion criteria (e.g., active suicidality/self-harm, severe alcohol, or substance use disorder) for the BC-CBT program overall are presented elsewhere.^23^ Clients must also have been assigned the digital activities specific to coping with the COVID-19 pandemic to be included for analysis. To allow a sufficient window of time to identify activity completions after assignment, any activities that were assigned 3 weeks or less before the end of the study period were excluded. Only the first completion of COVID-19 digital activities was included for analysis.

### Treatment

#### Blended care therapy overview

The BC-CBT program consists of sessions with a therapist, augmented by a curriculum of digital activities that are personalized by the therapist based on each client's presentation and needs. A total of 141 therapists were included in this study, including a variety of licensed mental health care professionals (clinical psychologists, marriage and family therapists, clinical social workers, and professional counselors), all of whom were extensively vetted for their proficiency with CBT through rigorous application reviews and clinical interviews. Digital activities are divided into *digital lessons*, which provide psychoeducation and skills training, and *digital exercises*, which are akin to digitized versions of typical CBT worksheets. Digital lessons and exercises are designed to work symbiotically, as the exercise facilitates independent practice of the skills and concepts presented in the lesson. In general, therapists orient clients to complete the digital lesson first, followed by its corresponding digital exercise. However, therapists have agency to decide which digital activity is assigned and in which order, depending on the clinical needs of the client, what has been covered in the therapy session, and what the client requests. Additional details about the BC-CBT program overall, as well as the provider-facing and client-facing therapy platforms, are presented elsewhere.^23^

#### “Coping with COVID-19” digital lesson and exercise

##### Development process

During late March 2020, as the pandemic was unfolding in the United States, BC-CBT therapists reported that their clients were experiencing exacerbated mental health symptoms. As such, the therapists highlighted the need for digital content in the BC-CBT platform that would specifically target COVID-19–related difficulties. Given the rapid and dynamic nature of the pandemic, timely development and dissemination of targeted therapeutic content were of utmost importance. To address this gap, Lyra's clinical team (three PhD clinicians and two LMFT clinicians) produced a digital lesson and a digital exercise teaching CBT skills to help address COVID-19–related distress.

As part of the development process, the team gathered targeted information by surveying BC-CBT providers (*n* = 64 respondents) about their experience working with clients during the COVID-19 pandemic (Appendix A1). The survey queried about the most concerning COVID-19–related issues reported by clients (e.g., getting sick, losing their jobs, and parenting challenges), the most frequently endorsed mental health difficulties (e.g., excessive fear and worry, rumination, difficulty sleeping, and problematic eating), as well as the CBT skills providers utilized most frequently to address these challenges (e.g., mindfulness, self-compassion, distress tolerance, and cognitive restructuring/thinking traps). Individual interviews were also conducted with experienced BC-CBT providers (two PhDs and two LMFTs) to allow for a more in-depth exploration of clinical needs and skills to target COVID-19 distress. Interviews followed a semistructured format and were conducted over video by a clinical psychologist. Interview content was informed by extrapolating common themes from the broader survey, and soliciting additional clinical input from interviewees. Recurrent themes across the surveys and interviews were then identified and incorporated into the digital activities.

##### Description of “Coping with COVID-19” lesson and exercise

The “Coping with COVID-19” lesson is presented through an animated 8-minute video using a storytelling approach, which has been found to be more relatable and normalizing for the viewers.^24^ Viewers follow a character on their therapy journey who is experiencing challenges due to the COVID-19 pandemic. As they work through these difficulties with the therapist in the lesson, the character learns core CBT skills to help manage the distress caused by the pandemic. Based on what the therapists reported in the surveys and individual interviews, a selected group of CBT skills were identified, such as identifying and normalizing certain emotions during the pandemic (e.g., feelings of loss and stress), differentiating between productive worry and unproductive worry, focusing more on what they can control, rather than what they cannot control, as well as identifying and challenging thinking traps (e.g., catastrophizing), judgmental thoughts, and unfair comparisons.

#### Measures

##### Acceptability and attitudes

After the video lesson, the client is asked to complete three questions on their attitudes regarding the lesson, specifically (1) relevance of the lesson to their COVID-19–related concerns, (2) their intent to apply the skills, and (3) perceived value of the lesson. These attitude responses are recorded on a 5-point Likert scale (Table 1).

**Table 1.** Coronavirus Disease 2019 Lesson Responses

Question	Response options	Responses (*n* = 377)	% of total responses (for each question)
Relevance: “This video covered approaches/skills that can help me better cope with the emotional impact of the COVID-19 pandemic”	Strongly agree	95	25.2
	Agree	238	63.1
	Neutral	39	10.3
	Disagree	5	1.3
	Strongly disagree	0	0.0
Intent: “I intend to apply approaches/skills from this video to my life”	Strongly agree	101	26.8
	Agree	231	61.3
	Neutral	41	10.9
	Disagree	4	1.1
	Strongly disagree	0	0.0
Feedback: “How valuable did you find the lesson?”	Extremely valuable	87	23.1
	Very valuable	149	39.5
	Moderately valuable	123	32.6
	Slightly valuable	15	4.0
	Not at all valuable	2	0.5
	No response	1	0.3

COVID-19, coronavirus disease 2019.

##### Coping skills use

The digital exercise instructs the client to select any and all approaches they used to cope with COVID-19 from a list of options (see Table 2 for a complete list). The options are subsumed under two separate categories: *active skills* (e.g., validating emotions) and *noticing* “thinking traps” (e.g., catastrophizing). The maximum possible number of skills selections was six; noticing “thinking traps” was coded as one skill (i.e., if any thinking trap option was selected, this counted as a positive selection of the noticing “thinking traps” skill). At the end of the exercise, the client is asked to rate how helpful they found these approaches in coping with COVID-19 on a 5-point Likert scale (ranging from “not at all helpful” to “extremely helpful”).

**Table 2.** Coronavirus Disease 2019 Exercise Responses

Coping skill text	Responses (*n* = 274)	% reporting skill use
In coping with the COVID-19 situation		
I validated my emotions (allowed myself to experience emotions such as anxiety, fear, and sadness)	201	73.4
I practiced (self-)compassion	128	46.7
I engaged in self-care (going for walks, eating healthy, etc.)	222	81.0
I brought myself back to the present moment	149	54.4
I focused more on what I control (versus on what I do not)	182	66.4
I noticed my mind was engaging in^a^	269	98.2
Catastrophizing: Imagining worst-case scenarios	175	63.9
Mind reading: Acting as if I knew what others were thinking	91	33.2
Unfair comparisons: Interpreting my performance based on unrealistic standards focusing on others doing better	161	58.8
Black and white thinking: Seeing a situation in extreme terms, all good or all bad	110	40.1
Unproductive worry	204	74.5
Self-judgments	192	70.1
Other thinking traps	57	20.8

^a^ These data report on the number of clients who made at least one thinking trap selection.

##### Completion rates

Completion rates were defined as the proportion of clients who were assigned the digital activity and completed it (at least once). Completion of the digital activities was verifiable through the Lyra platform, which logged time spent on video lessons and information provided in the exercises.

### Data analyses

#### Feasibility and acceptability data

To evaluate the feasibility and general use of the digital activities, the current study analyzed completion rates through descriptive statistics. To determine the acceptability of the digital activities, the frequency distributions of client responses to the attitude (relevance, intent, and value) questions were examined.

#### Group differences based on digital content assignment

Fisher's exact test or analysis of variance was used to determine differences in demographic and baseline symptom severity across clients who were assigned the digital video lesson only, the digital exercise only, and those who were assigned both.

#### Association between client attitudes and skills use

For clients who completed the lesson before the exercise, an ordinal logistic regression was used to analyze the association between client attitudes toward the lesson and the number of skills selected in the exercise. This analysis was selected due to the bounded range and bimodal distribution of the count outcome variable, with each number of selected skills treated as an ordinal category. The five ordinal response categories of the three attitude questions (Table 1) were converted to numeric scores ranging from 1 through 5, with higher numbers indicating more positive attitudes toward the lesson. They were then summed to create a composite “attitudes” variable, given that the three attitude questions were highly correlated with one another (*r*s = 0.70–0.85).

Ordinal logistic regression is based on the proportional odds assumption. This assumes that when the ordinal responses are split into two outcome groups (highest group vs. all lower groups, second highest group and up vs. remaining lower groups, etc.), the same coefficient for a predictor can be used to describe the relationship between all pairs of outcome groups. The proportional odds assumption was tested with the Brant test. Statistical significance was determined at *p* < 0.05. Descriptive statistics were conducted in Python 3.8 and the ordinal regression model was run in R 4.0.5.^25^

## Results

A total of 664 clients were assigned the “Coping with COVID-19” lesson and/or exercise. Table 3 reports the demographic information of the clients who were assigned the lesson and/or exercise. No group differences in demographic variables or baseline symptom severity were found between individuals who were assigned the lesson only, exercise only, or both.

**Table 3.** Demographics of Blended Care Therapy Clients

	Assigned lesson and exercise	Assigned lesson only	Assigned exercise only	*p*
Clients (*N*)	433	186	45	
Age (years), mean (SD)	33.9 (7.9)	34.4 (8.2)	35 (9.9)	*p* = 0.55
Gender, *n* (%)				
Female	315 (72.7)	124 (66.7)	27 (60.0)	*p* = 0.09
Male	117 (27.0)	62 (33.3)	18 (40.0)	
Unknown/did not answer	1 (0.2)	0 (0.0)	0 (0.0)	
Ethnicity, *n* (%)				
White	200 (46.2)	96 (51.6)	13 (28.9)	*p* = 0.17
Asian or Pacific Islander	109 (25.2)	47 (25.3)	14 (31.1)	
Other	93 (21.5)	32 (17.2)	11 (24.4)	
Prefer not to disclose/did not answer	31 (7.2)	11 (5.9)	7 (15.6)	
Highest education attained, *n (%)*				
College graduate or above	357 (82.4)	156 (83.9)	33 (73.3)	*p* = 0.67
Some college or AA degree	38 (8.8)	12 (6.5)	5 (11.1)	
High school graduate/GED or less	14 (3.2)	4 (2.2)	1 (2.2)	
Did not answer	24 (5.5)	14 (7.5)	6 (13.3)	
Baseline clinical status, mean (SD)				
GAD-7 score	10.1 (5.0)	10.2 (5.3)	8.4 (5.2)	*p* = 0.08
PHQ-9 score	8.6 (5.1)	8.7 (5.1)	7.3 (5.9)	*p* = 0.26

AA, associate in arts; GAD-7, generalized anxiety disorder-7; GED, graduate equivalency degree; PHQ-9, patient health questionnaire-9; SD, standard deviation.

A total of 619 clients were assigned the lesson, and 377 of those clients (60.9%) completed it. Upon lesson completion, the majority of clients reported high levels of agreement to the attitude (relevance, intent, and feedback) questions (Table 1). Specifically, 88.3% and 88.1% of the clients reported “Agree” or “Strongly agree” to the relevance and intent questions, respectively. In addition, 62.6% of the clients indicated that the lesson was at least “very valuable” on the feedback question.

A total of 478 clients were assigned the exercise, and 274 of those clients (57.3%) completed it. Upon exercise completion, 64.96% reported the exercise to be at least “moderately helpful.” The average number of selections made in the exercise was 4.3 (standard deviation = 1.4). Table 2 depicts the frequency of selections in the exercise.

A total of 433 clients were assigned both the lesson and exercise, representing 65.2% of all clients who were assigned either the lesson and/or exercise. For these clients, the completion rate was 60% for lessons, 56.8% for exercises, and 47.1% for both the lesson and exercise. Of those who completed both, 79.4% of clients completed the lesson before the exercise.

Among the 162 clients who completed the exercise after the lesson, an ordinal regression model found that the composite attitudes variable was a statistically significant predictor of the number of skills selected in the exercise (*β* = 0.21; standard error = 0.08; odds ratio [95% confidence interval] = 1.23 [1.06–1.44]; *p* < 0.01; reference group <2 selected skills). The proportional odds assumption was met under the Brant test (*p* = 0.09, the parallel regression assumption null hypothesis was not rejected). This suggests each 1-unit increase in the composite attitudes variable is associated with a 23.2% increase in the odds of selecting additional skills in the exercise.

## Discussion

As the pandemic progresses, the need for related content and skills will only increase, highlighting the need for scalable evidence-based interventions. To fill this gap, this study described the development of digital activities teaching cognitive-behavioral skills for coping with COVID-19 distress, and evaluated initial acceptability and feasibility data within a blended care model. As the majority of clients completed a digital activity if they were assigned it, this reflects the high need for and utility of pandemic-specific therapeutic content.

Among those who completed the lesson, there was a high level of agreement that (1) the content was relevant to their concerns, (2) they intended to practice the described approaches and skills, and (3) the lesson was valuable. For clients who completed the exercise after the lesson, higher agreement with these three attitude questions was positively associated with engagement in more skills (as indicated by the number of skill selections in the exercise). This suggests that clients who identify more strongly with the digital lesson content and commit to practicing these skills are more likely to actually implement the coping skills in their lives. These findings highlight the importance of optimizing a client's motivation to engage in these coping skills at the outset. By completing the lesson first, clients are able to better understand the context for the concepts and skills. In establishing this foundational knowledge, they may be more motivated to practice the skills in the digital exercise thereafter. More positive treatment attitudes and expectations have been linked to greater homework compliance and skills use.^20,21^

Ultimately, the development process for the “Coping with COVID-19” digital lesson and exercise can inform the development of other digital activities to address COVID-19–related distress using cognitive-behavioral skills. These activities were developed in direct response to emergent provider and client needs, necessitating swift action to address an unprecedented and universal surge in distress. Given the unique circumstances of the pandemic, these psychological tools had to be disseminated in a scalable, expedited, and safe manner. The strong acceptability and feasibility data reflect the utility of incorporating digital resources in psychotherapy, especially within the restrictive context of COVID-19. Ultimately, these findings underscore the impact of harnessing technology to disseminate these evidence-based interventions.

### Limitations

These results should be interpreted in the context of several limitations. First, given the within-person pilot nature of this study, we are unable to establish inferences of causality regarding the impact of the digital lesson on use of coping skills. However, completion of the exercise could have been impacted by some unmeasured variable (e.g., client motivation). Second, we did not measure the clients' actual practice of skills; as a result, it is possible that clients practiced the skill and did not report it (or did not practice the skill and reported that they did). Indeed, independent evaluation of homework completion is more accurate than patient self-report.^20^ Third, because we did not collect data on client mental health outcomes, we are unable to determine the association between completing the “Coping with COVID-19” digital activities and psychiatric symptoms. Fourth, our sample was fairly homogeneous, as they were relatively young (*M* age = 33 years), mostly employed, and at low risk (e.g., no significant substance use and no recent psychiatric hospitalizations). This intervention could have a different impact on older or unemployed individuals who may be more directly impacted by the pandemic.

### Future directions

As the nature of COVID-19 is changing, the psychological consequences of the pandemic and its mitigation efforts are similarly evolving. As a result, interventions addressing the distress related to COVID-19 will need to be flexible as well. To facilitate this dynamic process, further investigations should determine which components of the intervention should be adjusted, augmented, or prioritized at different time points, and with which subgroups of individuals. In addition, given that intention to practice skills predicted the number of skills reported, further work should be conducted to increase an individual's stated intention to practice therapy skills. Certain adjunctive interventions, such as motivational interviewing,^26^ could be added to augment motivation and intention to practice. Furthermore, although feedback was systematically gathered from treatment providers to solicit relevant content for the COVID-19–related digital activities, a comparable approach was not implemented for the clients themselves. As such, obtaining more in-depth feedback (e.g., surveys, interviews, and focus groups) directly from the clients would provide additional feedback regarding the feasibility, utility, and value of the activities, which would facilitate iterative improvements to the digital content. Taken together, as the United States grapples with COVID-19, considerable work is still needed to develop and evaluate interventions that can be quickly developed and deployed to a growing client base.

## Conclusion

By providing an overview of the process for developing digital activities targeting COVID-19–related distress, it is hoped to serve as a scalable model for creating and disseminating interventions specific to emergent events that have a population-level impact. It is especially vital to harness the advantages of technology within these contexts, as it significantly increases the accessibility of evidence-based interventions. Given the high acceptability and relevance of these activities, the utility of delivering cognitive-behavioral skills through a digital format is highlighted within a blended care model. Ultimately, targeted digital interventions for COVID-19 reduce provider burden and increase the accessibility of effective coping skills for clients within the pandemic.

## Abbreviations Used

BC-CBT: blended care CBT
CBT: cognitive-behavioral therapy
COVID-19: coronavirus disease 2019

## Appendix

### Appendix 1. Provider Survey on Experience Working with Therapy Clients on COVID-19–Related Concerns [Sample]

1. On average how much of a concern were the following COVID-19 issues for your clients? (use a scale of 0–4, 0 = not important, 1 = slightly importance, 2 = moderately important, 3 = important, 4 = very important)
  - Concerns about getting sick with COVID-19 themselves
  - Concerns about someone in their immediate family getting sick with COVID-19
  - Concerns about the impact of COVID-19 on their finances
  - Concerns around losing their jobs
  - Concerns about the impact of COVID-19 on their general health
  - Concerns around difficulties working from home
  - Concerns around parenting kids due to daycare/school closure
  - Difficulties due to feelings of isolation
  - Difficulties due to restricted range of physical activities
  - Worsening of chronic health problems
  - Other concern 1 _______
  - Other concern 2 ________
  - Other concern 3 _______
  - Other concern 4 ________
  - Other concern 5 _______
  - Other concern 6 ________
2. On average how often did your existing clients (not new to therapy) use interventions/skills they had learned in therapy to cope with distress from COVID-19 issues?
3. (0–4; 0 = never; 1 seldom; 2 = about half the time; 3 = usually; 4 = almost always)
3. On average how frequently did your clients experience the following psychological challenges due to COVID-19? (0–4; 0 = never; 1 seldom; 2 = about half the time; 3 = usually; 4 = almost always)
  a. Excessive fear and worry
  b. Rumination
  c. Difficulty sleeping
  d. Problematic eating
  e. Difficulty concentrating
  f. Increased use of alcohol, tobacco, or other drugs
  g. Increased use of other mood altering substances
  h. Worsening of chronic health problems
  i. Catastrophizing
  j. Depression/low mood
  k. Feeling overwhelmed
  l. Increased thoughts of suicide
  m. Decreased work productivity
  n. Feeling overwhelmed
  o. Self-criticism
  p. Excessively following media
  q. Other concern 1 _______
  r. Other concern 2 _______
  s. Other concern 3 _______
  t. Other concern 4 _______
  u. Other concern 5 _______
  v. Other concern 6 _______
4. How frequently did you focus with your clients in session on the following interventions to address COVID-19–related issues: (0–4; 0 = never; 1 = seldom; 2 = about half the time; 3 = usually; 4 = almost always)
  - Mindfulness
  - Relaxation
  - Gratitude techniques
  - Self-compassion
  - Values
  - Establishing routine and structure
  - Activating behaviors (0–10)
  - Normalizing, validating (0–10)
  - Sleep hygiene (0–10)
  - Communication skills with partners/family members who are sheltered in place together
  - General communication skills
  - Distress tolerance skills
  - Thinking traps/cognitive restructuring
  - Other intervention 1 _______ (0–10)
  - Other intervention 2 ________(0–10)
  - Other intervention 3 _______ (0–10)
  - Other intervention 4 ________(0–10)
  - Other intervention 5 _______ (0–10)
  - Other intervention 6 ________(0–10)
